# Longitudinal Assessment of Chest Computed Tomographic Findings After COVID-19 Infection

**DOI:** 10.7759/cureus.109771

**Published:** 2026-05-27

**Authors:** Miki Nishimori, Hitomi Iwasa, Kana Miyatake, Noriko Nitta, Takayasu Izumi, Rika Yoshimatsu, Tomohiro Matsumoto, Kazufumi Takamatsu, Akihito Yokoyama, Takuji Yamagami

**Affiliations:** 1 Department of Diagnostic and Interventional Radiology, Kochi Medical School, Kochi University, Nankoku, JPN; 2 Department of Respiratory Medicine and Allergology, Kochi Medical School, Kochi University, Nankoku, JPN

**Keywords:** chest computed tomography, covid-19 pneumonia, ct severity score (ctss), fibrotic-like changes, long-term progression

## Abstract

Background: The long-term evolution of fibrotic-like changes following moderate-to-severe COVID-19 pneumonia remains unclear. This study aimed to characterize the temporal progression of these abnormalities over a 12-month period.

Methods: This study was conducted as an ancillary analysis of a previously reported multicenter prospective observational study. From September 2020 to September 2021, we enrolled hospitalized adults (≥20 years old) with polymerase chain reaction- or antigen-confirmed COVID-19 classified as moderate or severe. A total of 83 patients who underwent chest CT at both admission and 12 months were included. All CT scans were independently reviewed by two board-certified radiologists. Fibrotic-like changes and CT severity scores (CTSS) were assessed at admission and at 3-, 6-, 9-, and 12-month follow-up. Clinical variables associated with fibrotic-like changes were analyzed.

Results: Fibrotic-like changes were present in 18/83 (21.7%) patients at admission and increased to 39/83 (47.0%) patients at 12 months. Their presence at admission was associated with older age, whereas their presence at 12 months was associated with older age and longer length of hospital stay. The prevalence of fibrotic-like changes increased significantly within the first three months (p<0.001) and remained stable thereafter. CTSS also increased significantly during the first three months (p=0.01), without further progression.

Conclusion: Fibrotic-like lung changes after COVID-19 pneumonia tend to progress mainly during the early post-hospitalization phase and stabilize by three months, with no substantial subsequent deterioration.

## Introduction

The COVID-19 pandemic caused by SARS-CoV-2 rapidly spread globally after its initial emergence in December 2019, resulting in widespread morbidity and mortality [1]. While the acute symptoms of COVID-19, such as fever, cough, and fatigue, have been extensively documented [2], the long-term pulmonary consequences remain incompletely understood.

Persistent fibrotic-like changes in the lungs of COVID-19 survivors have been demonstrated, often correlating with impaired respiratory function [3-5]. Chest CT has been instrumental in identifying these acute and chronic pulmonary abnormalities, with typical initial findings, including ground-glass opacity (GGO) and consolidations that evolve over time into reticular patterns suggestive of fibrosis [6,7]. However, significant gaps in knowledge remain regarding the longitudinal progression, stability, and clinical significance of these post-infectious fibrotic-like changes.

Most existing evidence is derived from retrospective single-center studies or studies with a limited follow-up duration. Prospective multicenter serial CT data are therefore needed to characterize their temporal evolution. In this multicenter prospective study, we aimed to evaluate the one-year longitudinal changes in pulmonary fibrotic-like lesions after COVID-19 infection. Specifically, we sought to determine whether fibrotic-like CT abnormalities progress or stabilize during the 12-month follow-up period and to explore their potential clinical relevance.

## Materials and methods

A subset of patients from a previously published cohort was included in this study [8]. While the earlier study primarily focused on clinical aspects, our analysis emphasized long-term imaging changes. All analyses were conducted independently to ensure methodological rigor and avoid redundancy.

Study design and participants

Fifty-five hospitals participated in this prospective cohort study. All were certified by the Japanese Respiratory Society and agreed before data were collected to participate in the study. Diagnosis of COVID-19 was based on SARS-CoV-2 polymerase chain reaction or antigen tests. Enrolled patients were hospitalized from February 2020 to September 2021 and were 20 years or older with moderate-to-severe COVID-19 pneumonia. Symptom severity was categorized as mild, moderate I, moderate II, or severe according to the guidelines of the Ministry of Health, Labour and Welfare [9]. Those indicated as mild had saturation of peripheral oxygen (SpO_2_) ≥ 96%, no respiratory symptoms or cough, and no shortness of breath. Moderate I patients (patients without respiratory failure) had 93% < SpO_2_ < 96%, with shortness of breath and pneumonia findings; moderate II patients (patients with respiratory failure) had SpO_2_ ≤ 93% and required oxygen administration; and the severe designation indicated admission to ICUs or mechanical ventilation. Patients were excluded if they were considered to have ethical conflicts or difficulties following the study protocol. Patient information was retrieved from electronic case records.

CT imaging and evaluation

Chest CT scans were performed in the supine position during end-inspiration without contrast. Other imaging protocols followed institution-specific standards. In the original multicenter cohort, follow-up CT assessments were conducted by attending physicians or radiologists at each participating institution. For the purpose of the present sub-analysis, however, all CT scans were re-evaluated independently by two board-certified radiologists at our institution. The radiologists were blinded to all clinical information, including disease severity, laboratory data, and clinical outcomes. Any discrepancies were resolved by consensus. This centralized and blinded re-evaluation was performed to ensure consistent application of imaging criteria and to minimize inter-facility variability inherent in the original multicenter assessments. Based on the most significant proportion of lung abnormalities, the predominant CT pattern was categorized as GGO, consolidation, or reticulation. Recorded CT findings included nodule/mass, pleural effusion, interlobar pleural traction, traction bronchiectasis, honeycombing, and parenchymal bands [10]. The presence of traction bronchiectasis, honeycombing, or parenchymal bands defined fibrotic-like changes. A semiquantitative CT score was calculated as the CT severity score (CTSS) for each of the five lobes (0 to 5), with a maximum total score of 25 [11].

Follow-up visits

If the patient’s doctor determined that the results of imaging, as well as signs and symptoms, did not indicate abnormalities, follow-up ended. However, if any of the clinical findings, CT results, or results of pulmonary function, blood tests, or other relevant tests did not improve, patients were followed up every 3 months until 12 months. All data collected were retrieved from the electronic data capture system of the Department of Data Management at the Integrated Center for Advanced Medical Technologies, Kochi Medical School Hospital. Based on the study protocol, data were collected from 932 patients at 3 months, 491 at 6 months, 251 at 9 months, and 125 at 12 months. Therefore, the number of patients who underwent follow-up CT examinations differed at each time point. We only included patients who underwent both chest CT imaging at admission and 12 months post-admission.

Investigated parameters

We analyzed longitudinal changes in CT findings and examined the associations between fibrotic-like changes observed on CT at hospitalization and 12 months and relevant clinical parameters. Furthermore, we evaluated the progression of fibrotic-like changes and the trajectory of CTSS over 12 months in a subgroup of 38 patients with complete serial CT data from admission to 12 months.

Statistical analysis

Statistical analyses were conducted using SPSS version 29.0 (IBM Corp., Armonk, NY). Logistic regression was used for univariate and multivariate analyses. Missing values were handled using listwise deletion. Cochran’s Q and Friedman tests were applied to repeated measures data for nominal and ordinal/continuous variables, respectively. Bonferroni correction was used for multiple comparisons, with p<0.05 considered statistically significant. Variables showing a statistically significant association in the univariate analysis were entered into the multivariate logistic regression model.

## Results

**Figure 1:**
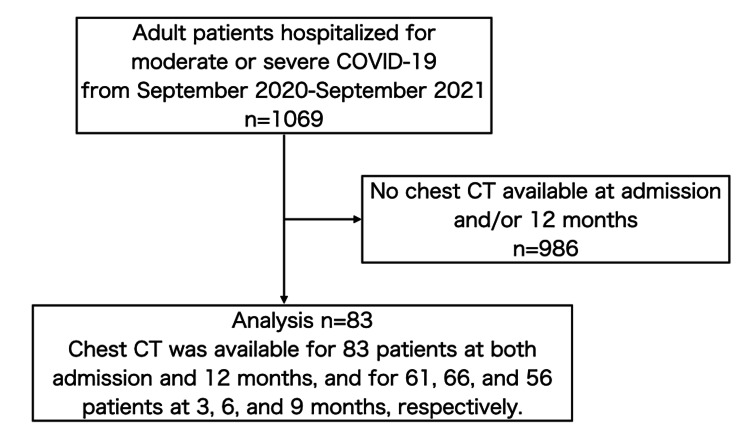
Flowchart of patient selection Of the 1,069 patients initially enrolled, patients without follow-up chest CT at 12 months or with incomplete imaging data were excluded. Ultimately, 83 patients who underwent chest CT both at hospitalization and 12 months were included in the primary analysis.

Among the 1,069 patients initially screened, 83 (8%) were finally enrolled in this analysis (Figure 1).

Table 1 shows the baseline and 12-month post-admission characteristics of the enrolled patients. At baseline, 18 (21.7%) patients exhibited fibrotic-like changes on chest CT. In the univariate analysis at baseline, only age was significantly different (p<0.05); therefore, a multivariate analysis was not performed. At 12 months post-admission, fibrotic-like changes were observed in 39 of 83 (47.0%) patients. In the univariate analysis at 12 months post-admission, age, history of heart disease, serum lactate dehydrogenase and albumin levels, and length of hospital stay (LOS) were significantly associated with fibrotic-like changes on chest CT (p<0.05). These variables were subsequently included in a multivariate analysis, which identified age and LOS as independent factors significantly associated with fibrotic-like changes (p<0.05).

**Table 1:** Baseline and 12-month post-admission characteristics of the 83 enrolled patients ALB, albumin; BT, body temperature; CRP, C-reactive protein; CTSS, CT severity score; DM, diabetes mellitus; KL-6, Krebs von den Lungen-6; LDH, lactate dehydrogenase; LOS, length of stay. Data are presented as n (%) or mean (± standard deviation), as appropriate. *p-value < 0.05 indicates a statistically significant difference. Numbers in parentheses indicate the number of patients with available data for each variable. The total study population was 83 patients; some variables had missing data, resulting in smaller denominators.

Characteristics	No fibrotic-like change (zero month/baseline) (n=65)	Fibrotic-like change (zero month/baseline) (n=18)	Univariable p-value	No fibrotic-like change (12 months post-admission) (n=44)	Fibrotic-like change (12 months post-admission) (n=39)	Univariable p-value	Multivariable p-value
Disease severity (severe) - n (%)	32 (49.2)	8 (44.4)	0.719	17 (38.6)	23 (59.0)	0.066	Not included
Age - mean ± SD	66±11	72±12	0.042*	64±11	70±11	0.021*	0.041*
Sex (male) - n (%)	51 (78.5)	11 (61.1)	0.14	33 (75.0)	29 (74.4)	0.947	Not included
Smoking history (yes) - n (%)	40 (62.5/n=64)	10 (55.6/n=18)	0.594	27 (62.8/n=43)	23 (59.0/n=39)	0.724	Not included
Body mass index (kg/m^2^) - mean ± SD	25.1±3.5 (n=61)	25.0±3.9 (n=17)	0.895	25.1±3.5 (n=41)	25.1±3.8 (n=37)	0.96	Not included
Underlying disease - n (%)							Not included
Heart	8 (12.3)	4 (22.2)	0.297	3 (6.8)	9 (23.1)	0.046*	0.322
Brain	4 (6.2)	0 (0)	0.999	2 (4.5)	2 (5.1)	0.902	Not included
Respiratory	8 (12.3)	3 (16.7)	0.631	6 (13.6)	5 (12.8)	0.913	Not included
Lipidemia	20 (30.8)	5 (27.8)	0.807	17 (38.6)	8 (20.5)	0.076	Not included
DM	19 (29.2)	3 (16.7)	0.292	12 (27.3)	10 (25.6)	0.867	Not included
Liver	3 (4.6)	1 (5.6)	0.869	3 (6.8)	1 (2.6)	0.385	Not included
Malignancy	10 (15.4)	1 (5.6)	0.298	8 (18.2)	3 (7.7)	0.171	Not included
BT - mean ± SD	37.7±1.1	37.6±1.2	0.812	37.6±1.1	37.6±1.1	0.996	Not included
SpO_2_ - mean ± SD	92±8 (n=64)	92±4 (n=18)	0.881	93±4 (n=43)	91±10 (n=39)	0.352	Not included
Mechanical ventilation (yes) - n (%)	21 (32.3)	5 (27.8)	0.714	10 (22.7)	16 (41.0)	0.076	Not included
CRP - mean ± SD	9.9±7.2	10.9±13.6	0.68	9.2±6.5	11.2±11.0	0.301	Not included
LDH - mean ± SD	395±115 (n=65)	465±268 (n=17)	0.128	374±104 (n=44)	452±199 (n=38)	0.037*	0.36
KL-6 - mean ± SD	411±302 (n=44)	529±392 (n=8)	0.343	425±353 (n=30)	434±267 (n=22)	0.92	Not included
ALB - mean ± SD	3.1±0.6 (n=61)	3.0±0.9 (n=18)	0.576	3.3±0.5 (n=40)	2.9±0.8 (n=39)	0.006*	0.191
LOS - mean ± SD	31±26	33±27	0.732	22±15	43±31	0.001*	0.025*
CTSS - mean ± SD	15±6	17±5	0.15	14±6	17±6	0.077	Not included

In the imaging analysis, GGO was the most common finding at hospitalization and transitioned to reticulation as the predominant feature at 12 months. Compared with findings at hospitalization, traction bronchiectasis, honeycombing, and parenchymal bands showed a marked increase at 12 months (Table 2). Because follow-up CT examinations were not available for all patients at each time point, the number of patients evaluated differed across follow-up time points.

**Table 2:** Chest CT findings of COVID-19 pneumonia during one-year follow-up Data are shown as N (%). The number of evaluated patients varied across follow-up time points due to incomplete availability of follow-up CT examinations.

CT feature	At hospitalization (n=83)	3-month CT (n=61)	6-month CT (n=66)	9-month CT (n=56)	12-month CT (n=83)
Predominant lesion					
Ground-glass opacities	65 (78.3)	37 (60.7)	30 (45.5)	24 (42.3)	33 (40)
Consolidation	18 (21.7)	0 (0)	0 (0)	0 (0)	0 (0)
Reticulation	0 (0)	22 (36.1)	34 (51.5)	31 (55.4)	42 (50.6)
Normal	0 (0)	2 (3.28)	2 (3.03)	1 (1.79)	8 (9.64)
Nodule/mass	4 (4.8)	0 (0)	1 (1.52)	0 (0)	0 (0)
Pleural effusion	10 (12.0)	0 (0)	1 (1.52)	1 (1.79)	1 (1.20)
Interlobar pleural traction	6 (7.2)	6 (9.84)	9 (13.6)	9 (16.1)	11 (13.3)
Traction bronchiectasis	16 (19.3)	18 (29.5)	24 (36.4)	22 (39.3)	30 (36.1)
Honeycomb	8 (9.6)	8 (13.1)	11 (16.7)	11 (19.6)	15 (18.1)
Parenchymal bands	8 (9.6)	23 (37.7)	20 (30.3)	20 (37.1)	27 (32.5)

**Figure 2:**
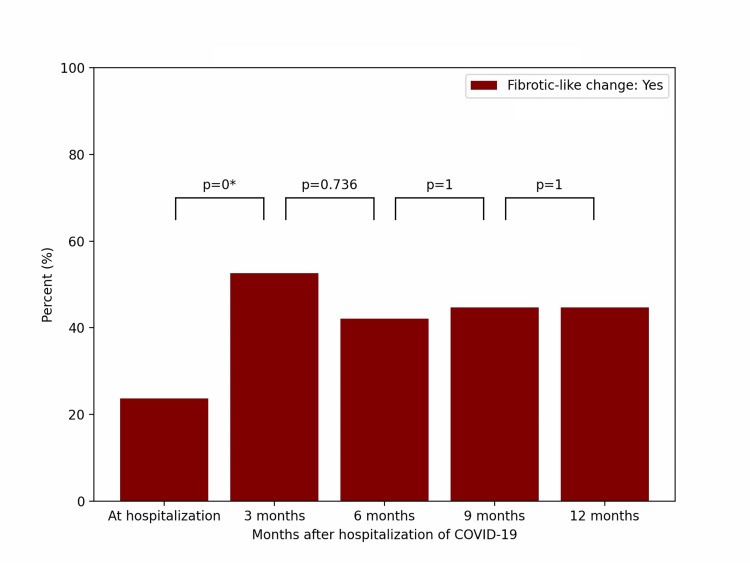
Fibrotic-like changes over time In a subset of 38 patients with complete serial CT data, fibrotic-like changes significantly increase during the first three months after hospitalization (p<0.001) and subsequently appeared to plateau.

**Figure 3:**
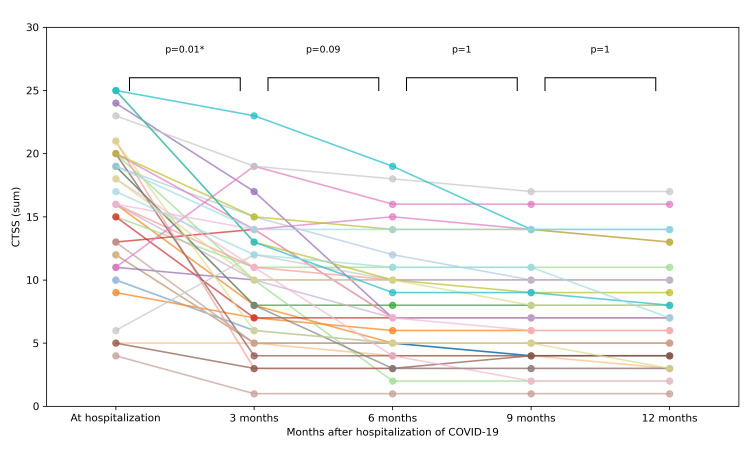
Changes in CTSS over time CTSS (sum) represents the sum of semiquantitative scores assigned to each of the five lung lobes, ranging from 0 to 25 and reflecting the overall extent of pulmonary involvement. CTSS declined significantly during the early phase (p=0.01), with no substantial changes observed thereafter. CTSS, CT severity score.

Furthermore, in the subset of 38 patients with complete serial CT data, fibrotic-like changes increased significantly during the first three months after hospitalization (p<0.001) and subsequently plateaued (Figure 2). Similarly, the CTSS declined significantly during the early phase (p=0.01), with no substantial changes observed thereafter (Figure 3).

**Figure 4:**
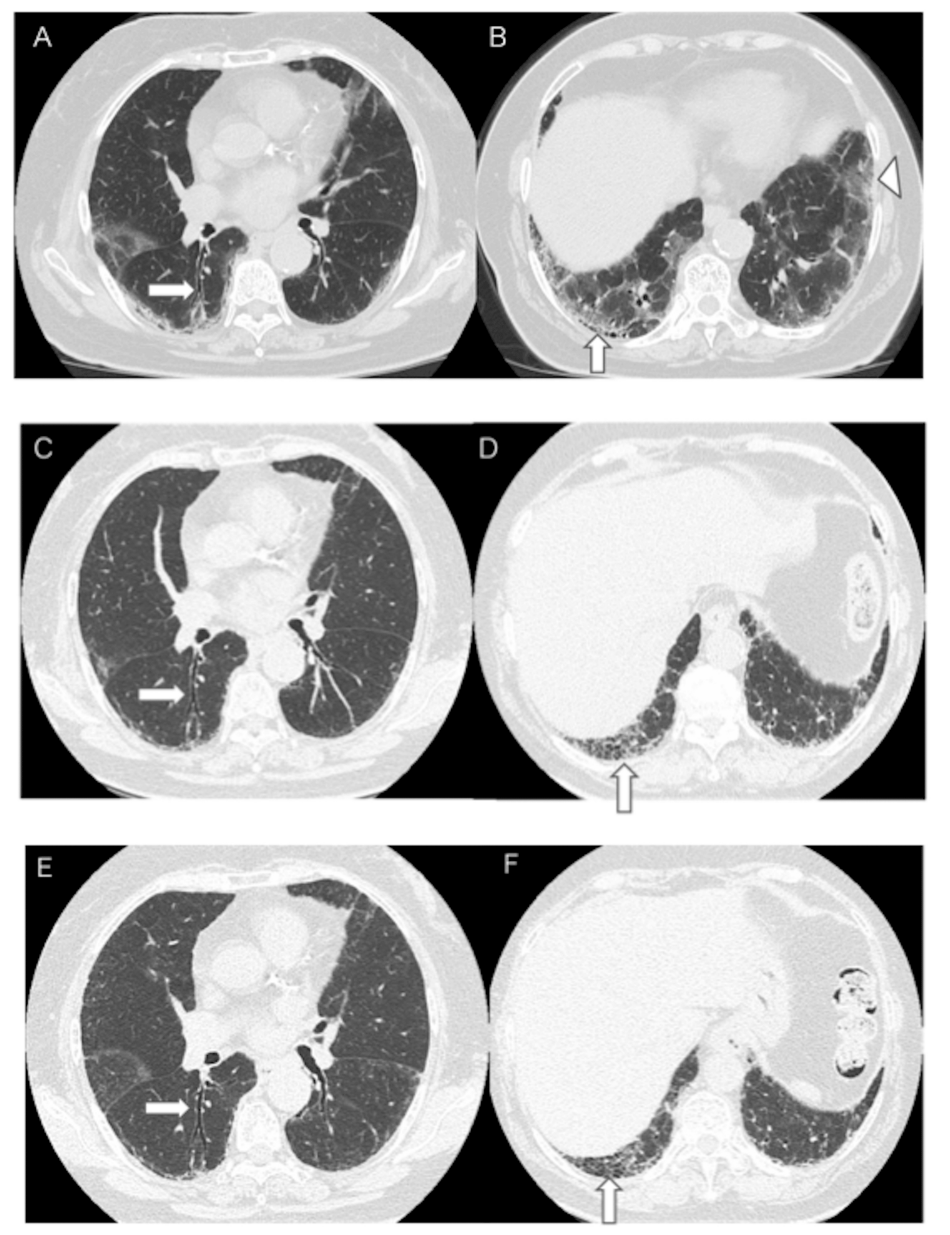
Representative longitudinal chest CT findings in an 84-year-old woman after hospitalization for COVID-19 (A, B) CT images obtained at hospitalization show extensive ground-glass opacity as the predominant pattern, with traction bronchiectasis (A, arrow) and honeycombing (B, arrow). The CT severity score (CTSS) was 19. (C, D) At three months after hospitalization, the predominant pattern had changed from ground-glass opacity to reticulation. Traction bronchiectasis (C, arrow) and honeycombing (D, arrow) remained visible. The CTSS decreased to 14. (E, F) At 12 months after hospitalization, reticulation remained the predominant pattern, with persistent traction bronchiectasis (E, arrow) and honeycombing (F, arrow). The CTSS remained 14.

Figure 4 illustrates the progression of typical chest CT findings following COVID-19 infection.

## Discussion

This study provides prospective multicenter evidence suggesting that fibrotic-like changes may increase during the early post-acute phase and subsequently stabilize from approximately three months after hospitalization for COVID-19 in a selected cohort. Han et al. demonstrated that fibrotic interstitial lung abnormalities remained stable from six months to two years after COVID-19 pneumonia [12]. However, our results suggest that stabilization may begin earlier, potentially within the first three months after hospitalization. Bocchino et al. similarly reported that fibrotic changes were detected between three and six months and remained stable at one year [5]. While their findings are consistent with ours, they did not statistically evaluate the progression.

Our analysis identified advanced age and LOS as independent clinical risk factors associated with fibrotic-like pulmonary changes following COVID-19 pneumonia. These findings align with previous research [13,14]. Age was significantly associated with fibrotic-like changes observed both at hospital admission and at 12 months post-admission in our study. In a study conducted by Xu et al. using a mouse model, it was reported that, in addition to increases in inflammatory and fibrotic factors following injury, increased mobilization of fibrocytes might contribute to age-related susceptibility to pulmonary fibrosis [15]. Such findings suggest the importance of early identification of patients who are at higher risk for pulmonary fibrosis after COVID-19 infection, underscoring the necessity for long-term follow-up and rehabilitation interventions after discharge. However, our study did not find significant associations between several previously reported clinical factors, including disease severity, sex, smoking history, and pre-existing respiratory conditions, and persistent fibrotic-like changes at 12 months post-admission [13]. This discrepancy may partly result from patient selection bias in our cohort. Further investigation is required to confirm and clarify these relationships.

At hospital admission, fibrotic-like changes were detected on CT imaging in 18 of 83 patients (21.7%). Although studies focusing on early-stage fibrotic-like changes in COVID-19 pneumonia remain limited, our findings align with those of Fang et al., who also observed these changes at hospitalization [16]. Previous research suggested that CT abnormalities peak approximately 10 days after symptom onset, followed by improvement [17]. Li et al. reported that the most common manifestation histologically was diffuse alveolar damage (DAD) in 28 (93.3%) cases, which showed predominantly acute (32%), organizing (25%), and/or fibrosing (43%) patterns in COVID-19 infection [18]. Following an initial inciting injury, DAD progresses through three distinct phases: the acute phase occurring within the first 7-10 days, the organizing phase commencing thereafter, and the fibrosing phase developing approximately three weeks post-injury [19]. The majority of patients who survive DAD will have permanent abnormalities shown on follow-up CT [20]. CT findings that can be observed in patients after DAD include persistent abnormalities such as fibrotic-like changes, as in this study. The fibrotic-like changes observed in the early stages, such as within three months, may represent the progression of fibrosis associated with DAD.

Compared with our CT findings at hospitalization, the frequency of reticulation increased significantly at 12 months. Finally, only eight (9.64%) patients exhibited normal CT findings, consistent with previous studies showing the persistence of imaging abnormalities following COVID-19 pneumonia [21,22]. At hospitalization, GGOs were the predominant CT findings; however, at 12 months, reticulation frequency increased, but some GGOs also persisted. These findings align with those reported in previous studies, supporting our observations. Persistent GGO and reticulation potentially reflect irreversible microscopic interstitial abnormalities. The absence of correlation between the initial CTSS and fibrotic-like changes at 12 months could be attributed to the fact that CTSS in the acute phase primarily captured reversible inflammatory changes rather than the progression of fibrosis itself. Thus, acute-phase CTSS may indicate the extent of inflammation, but it does not necessarily predict the final fibrotic outcome. These results highlight the complexity in distinguishing acute reversible lung changes from chronic potentially irreversible fibrotic changes, emphasizing the limitations inherent in relying solely on CT severity scoring systems during the acute stage of COVID-19.

This study has several limitations. First, the primary analysis included only patients who underwent CT both at admission and 12 months, which may have introduced selection bias and overestimated the prevalence and stability of fibrotic-like changes. Second, although CT images were blindly re-evaluated by two board-certified radiologists, the original scans were acquired across multiple institutions using heterogeneous protocols, including differences in acquisition parameters and reconstruction settings; this may have affected image quality and the reproducibility of CT-based assessments. Third, pulmonary function tests, symptoms, and long-term clinical outcomes were not evaluated in this study, preventing assessment of the physiological and clinical relevance of radiological stability. Finally, the study period spanned multiple variant waves and evolving treatment strategies, which could not be analyzed but may have influenced imaging findings.

## Conclusions

Fibrotic-like changes in the lungs following COVID-19 pneumonia tend to stabilize in frequency within approximately three months and persist over the long term. These findings suggest that most structural lung alterations may occur early after acute infection, with limited progression thereafter. Early identification of patients at risk may therefore be important for optimizing follow-up strategies and post-discharge management, while unnecessary long-term imaging in low-risk patients might be avoided.
